# Reconstruction of the biosynthetic pathway for the core fungal polyketide scaffold rubrofusarin in *Saccharomyces cerevisiae*

**DOI:** 10.1186/1475-2859-12-31

**Published:** 2013-04-04

**Authors:** Peter Rugbjerg, Michael Naesby, Uffe H Mortensen, Rasmus JN Frandsen

**Affiliations:** 1 Evolva SA, Duggingerstrasse 23, Reinach CH-4153, Switzerland; 2 Søltofts Plads Building 223, Kgs Lyngby DK-2800, Denmark

**Keywords:** Iterative polyketide synthase, Pigment, Heterologous production, YWA1, Nor-rubrofusarin, Aurofusarin

## Abstract

**Background:**

Fungal polyketides include commercially important pharmaceuticals and food additives, e.g. the cholesterol-lowering statins and the red and orange monascus pigments. Presently, production relies on isolation of the compounds from the natural producers, and systems for heterologous production in easily fermentable and genetically engineerable organisms, such as *Saccharomyces cerevisiae* and *Escherichia coli* are desirable. Rubrofusarin is an orange polyketide pigment that is a common intermediate in many different fungal biosynthetic pathways.

**Results:**

In this study, we established a biosynthetic pathway for rubrofusarin in *S. cerevisiae*. First, the *Fusarium graminearum* gene encoding polyketide synthase 12 (*PKS12*) was heterologously co-expressed with the *Aspergillus fumigatus* gene encoding phosphopantetheinyl transferase (*npgA*) resulting in production of YWA1. This aromatic heptaketide intermediate was converted into nor-rubrofusarin upon expression of the dehydratase gene *aurZ* from the aurofusarin gene cluster of *F. graminearum.* Final conversion into rubrofusarin was achieved by expression of the O-methyltransferase encoding gene *aurJ*, also obtained from the aurofusarin gene cluster, resulting in a titer of 1.1 mg/L. Reduced levels of rubrofusarin were detected when expressing *PKS12*, *npgA*, and *aurJ* alone, presumably due to spontaneous conversion of YWA1 to nor-rubrofusarin. However, the co-expression of *aurZ* resulted in an approx. six-fold increase in rubrofusarin production.

**Conclusions:**

The reconstructed pathway for rubrofusarin in *S. cerevisiae* allows the production of a core scaffold molecule with a branch-point role in several fungal polyketide pathways, thus paving the way for production of further natural pigments and bioactive molecules. Furthermore, the reconstruction verifies the suggested pathway, and as such, it is the first example of utilizing a synthetic biological “bottom up” approach for the validation of a complex fungal polyketide pathway.

## Background

Polyketides exhibit large structural diversity and have attracted considerable interest from the pharmaceutical industry as they form a chemical class rich in bioactive molecules. The biological activities of fungal polyketides span from potent carcinogenic toxins (aflatoxin) over immunosuppressants (dalesconols), anti-tumor compounds (aspergiolide A) to block-buster cholesterol-lowering drugs (statins) [1-3]. In addition, many fungal polyketides also display vibrant colors due to their polyaromatic structures, which have made them attractive for the textile and food industries as natural dyes [4,5].

**Figure 1:**
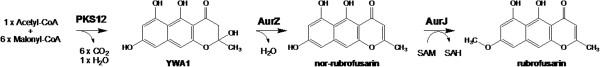
**The rubrofusarin biosynthetic pathway.** Biosynthetic pathway of rubrofusarin in *F. graminearum* established by targeted gene deletion experiments [12,18].

Fungal polyketides of commercial interest are often produced in nature by species that are not easily cultured in standard liquid fermentation setups, due to their filamentous growth, and formation of dense mycelium clumps resulting in increased viscosity and reduced oxygen solubility. The transfer of such biosynthetic pathways to standard industrial cell factories, such as *Saccharomyces cerevisiae*, could offer an attractive alternative as it would allow easier fermentation and process optimization via the existing metabolic engineering strategies. However, only a few examples of successful heterologous expression of genes encoding fungal polyketide synthases in yeast have been reported in literature. These include the 6-MSA synthase gene from *Penicillium patulum* resulting in 6-methylsalicylic acid (6-MSA) production [6-8]*, rdc1*/*rdc5* from *Pochonia chlamydosporia*[9] resulting in *(R)*-monocillin II production, and *hpm3/hpm8* resulting in hypothemycin production [10]. Most recently the individual expression of three PKSs from *Chaetomium globosum* and one from *Coprinopsis cinerea* was reported in an effort to characterize orphan PKSs [11]. These results show that yeast heterologous expression can provide an avenue for production of industrially relevant polyketides and for characterizing orphan polyketide synthase genes from non-model organisms. However, common for these examples is that the studies only aimed to produce the first stable intermediate in the respective biosynthetic pathway and have not included the heterologous expression of any tailoring enzymes responsible for modification of the synthase product. To test the feasibility of using yeast for production of complex polyketides we aimed to reconstruct a fungal polyketide pigment biosynthetic pathway, which leads to the formation of rubrofusarin (Figure 1). Rubrofusarin is an orange-brown pigment produced by a wide range of different fungal species, such as *Fusarium graminearum, Aspergillus niger, Aspergillus parasiticus* and *Ustilaginoidea virens*[12-14]. The biological activity of rubrofusarin is reported to include inhibition of human DNA topoisomerase II-α [15] and general antibiotic effects on *Mycobacterium tuberculosis* and various filamentous fungi [16]. Interestingly, the fate of this polyketide core scaffold differs in the particular fungal species due to their unique repertoires of tailoring enzymes, resulting in production of such different end products as rubrofusarin B, aurofusarin, nigerone, nigerasperone A, chaetochromin, ustilaginoidin and parasperone A [12,17].

Currently, the best described biosynthetic pathway, which includes rubrofusarin as an intermediate, is the aurofusarin pathway in *F. graminearum*. In this species, the first stable pathway intermediate on the path to rubrofusarin is the yellow naphthopyrone YWA1, which is synthesized by the condensation of one acetyl-CoA and six malonyl-CoA molecules, catalyzed by the type I iterative non-reducing PKS12 [18]. Similar reactions have been reported for *Aspergillus nidulans*[19]. The formed YWA1 is dehydrated by AurZ to form the orange pigment nor-rubrofusarin, which is then O-methylated by AurJ to yield rubrofusarin (Figure 1) [12,18]. Polyketide biosynthetic pathways are typically encoded by gene clusters in fungi and mapping of the aurofusarin pathway in *F. graminearum* has therefore primarily been based on targeted deletion of the genes surrounding *PKS12*[12,18,20]. Thus, the possibility exists that genes located elsewhere in the genome might be directly involved in the formation of rubrofusarin. Hence, an appropriate bottom-up proof of the sole involvement of *PKS12*, *aurZ* and *aurJ* in rubrofusarin formation would be their heterologous expression in a phylogenetically distant organism.

Heterologous production in *S. cerevisiae* of other important compound classes with a complex biosynthetic origin, e.g. terpenoids [21] and plant type III polyketides [22], has already been well established. To provide a proof-of-concept that this approach is also a viable option for polyketide pathways with a fungal origin, and to prove the proposed biosynthetic pathway, we here present the stepwise construction of a rubrofusarin pathway in the heterologous host *S. cerevisiae*.

### Results and discussion

Rubrofusarin has previously been described to have antifungal properties, and to avoid problems with toxic effects of intermediates or the end-product during strain construction, the open reading frame (ORF) encoding the wild type PKS12 was placed under control of the inducible *CUP1* promoter to allow control of expression onset and strength. Codon optimized ORFs for *aurZ, aurJ* and *npgA* were expressed using unique constitutive promoters and terminators (Table 1). The genes were inserted individually into different plasmids, derived from pRS413-6 [23], each carrying unique auxotrophic selection marker genes along with the unique promoter and terminator elements. This plasmid-based approach was chosen to allow for easy construction of any desired combination of genes in *S. cerevisiae* to study the sequential order of individual catalytic steps in the biosynthetic pathway (Table 1).

**Table 1:** ***S. cerevisiae *strains used in the study**

**Strain**	**Plasmids carried**	**Relevant genotype**
*S. cerevisiae* control	pRS416 (*URA3)*	-
	pRS413 (*HIS3)*	
	pRS414 (*TRP1*)	
	pRS415 (*LEU2)*	
*S. cerevisiae PKS12/npgA*	pRS416::p_CUP1_-***PKS12***-t_ADH1_,	*PKS12, npgA*
	pRS413::p_PYK1_-***npgA***-t_TEF1_	
	pRS414	
	pRS415	
*S. cerevisiae PKS12/npgA/aurZ*	pRS416::p_CUP1_-***PKS12***-t_ADH1_,	*PKS12, npgA, aurZ*
	pRS413::p_PYK1_-***npgA***-t_TEF1_	
	pRS414::p_TEF1_-***aurZ***-t_ENO2_	
	pRS415	
*S. cerevisiae PKS12/npgA/aurJ*	pRS416::p_CUP1_-***PKS12***-t_ADH1_,	*PKS12, npgA, aurJ*
	pRS413::p_PYK1_-***npgA***-t_TEF1_	
	pRS415::p_GPD1_-***aurJ***-t_CYC1_	
	pRS414	
*S. cerevisiae PKS12/npgA/aurZ/aurJ*	pRS416::p_CUP1_-***PKS12***-t_ADH1_,	*PKS12, npgA, aurZ, aurJ*
	pRS413::p_PYK1_-***npgA***-t_TEF1_	
	pRS414::p_TEF1_-***aurZ***-t_ENO2_	
	pRS415::p_GPD1_-***aurJ***-t_CYC1_	

*S. cerevisiae* strains that were analyzed for production of rubrofusarin and precursors. Final plasmids transformed into *S. cerevisiae* strains showing the vector backbone in which the constructed promoter (p) – ORF – terminator (t) gene cassettes were inserted. The auxotrophic marker gene is also shown. All plasmids were maintained by the low copy number sequences ARS/CEN.

#### Heterologous production of YWA1

**Figure 2:**
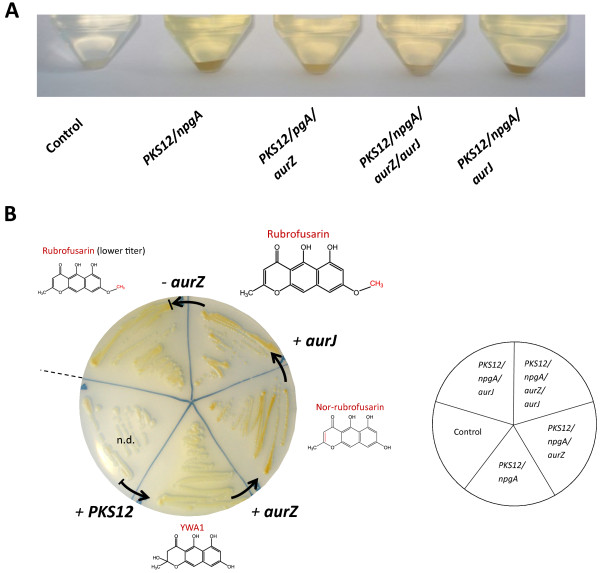
**Phenotype of the constructed *S. cerevisiae *strains.** Cultures of *S. cerevisiae* strains, carrying the genes shown, were grown in **A**) SC medium with 300 μM CuSO_4_ and without uracil, histidine, tryptophan and leucine. Cells were grown for 4 days at 30°C, 160 rpm shaking, and collected by centrifugation. **B**) SC agar plates with 500 μM CuSO_4_ and without uracil, histidine, tryptophan and leucine. Grown for 4 days at 30°C. The strains accumulated the indicated rubrofusarin pathway products.

**Figure 3:**
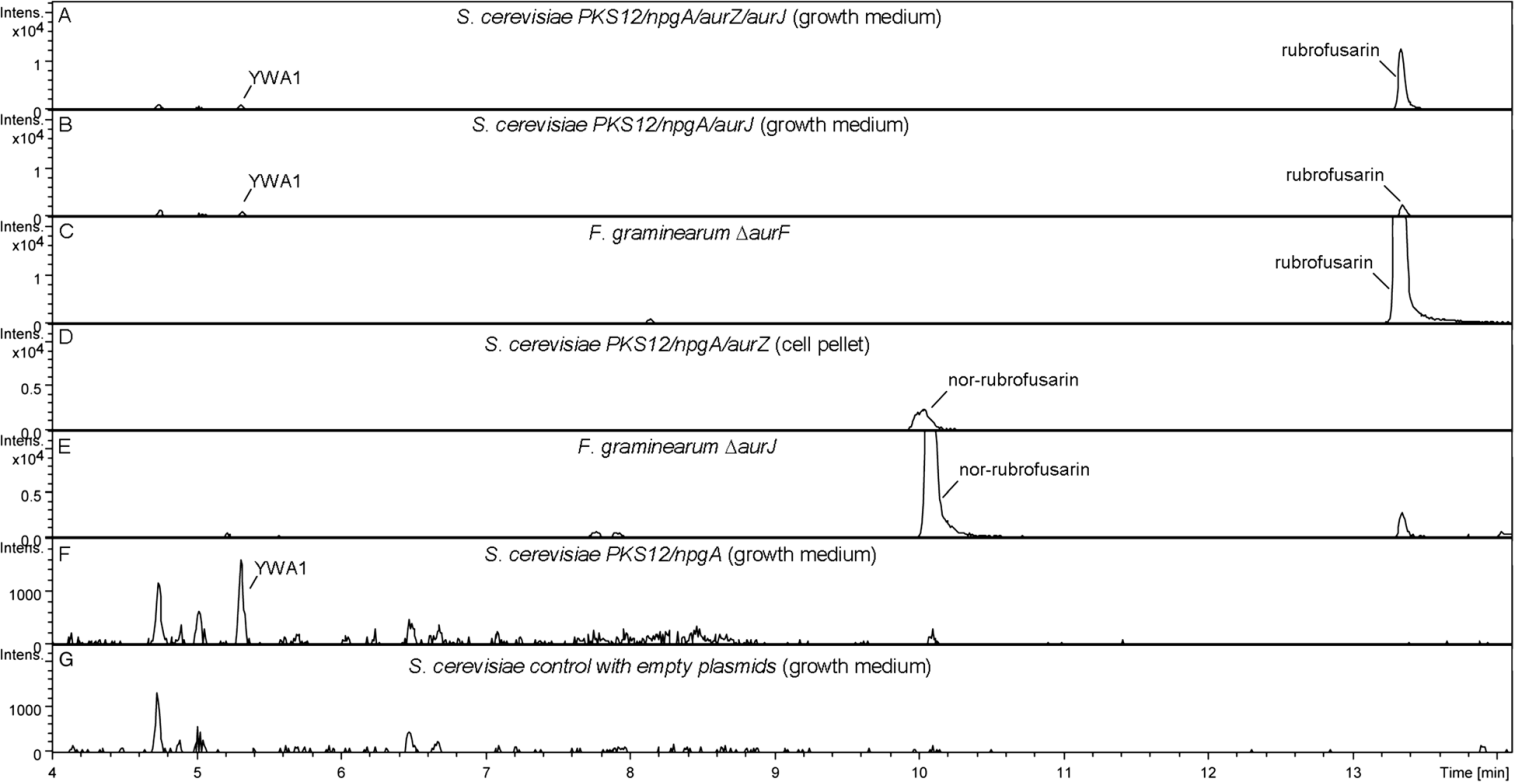
**Chemical analysis of strains.** Extracted ion chromatograms (EIC) for the analyzed strains. EIC for **A**) *S. cerevisiae PKS12/npgA/aurZ/aurJ*, growth medium (GM), **B**) *S. cerevisiae PKS12/npgA/aurJ,* (GM), **C**) rubrofusarin-accumulating reference *F. graminearum ΔaurF*, **D**) *S. cerevisiae PKS12/npgA/aurZ*, cell pellet, **E**) nor-rubrofusarin-accumulating reference *F. graminearum ΔaurJ*, **F**) *S. cerevisiae PKS12/npgA* (GM), **G**) Control *S. cerevisiae* strain with empty plasmids (GM). Note that the scales on the Y-axes are not identical between the different chromatograms.

Expression of *PKS12* and *npgA* in *S. cerevisiae* resulted in the production of a yellow pigment which was not seen in the control strain carrying empty plasmids. In contrast to plates without added copper, yellow-colored pigment was visually observed after approx. 24 hours of cultivation on synthetic complete (SC) agar plates supplemented with copper. Following incubation for an additional 2-3 days, a yellow pigment became visible in the medium surrounding the *S. cerevisiae* colonies, indicating that the produced pigment was escaping from the cells. In SC liquid cultures a yellow color became visually observable after 2-3 days of cultivation (Figure 2). Chemical analysis, using LC-MS, of the growth medium from the induced *PKS12*/*npgA* strain revealed a new peak with a retention time (RT) of 5.3 min and a [M + H]^+^ of 277.07 m/z. Based on the exact mass, the compound was identified as the pigment YWA1 (Figure 3). The intensity of the peak was, however, relatively weak suggesting either a low production level, poor extraction or ionization of YWA1, or rapid degradation as was previously reported by Fujii and colleagues working with YWA1 in *A. oryzae*[24]. This compound is also known from *Aspergillus fumigatus* in which it is converted to 1,8-dihydroxynaphthalene (DHN) in multiple steps, including a chain-shortening reaction catalyzed by Ayg1 [25], and in *A. nidulans* where it is the precursor for the green conidial pigment [19].

### Heterologous production of nor-rubrofusarin

Introduction of the dehydratase-encoding *aurZ* gene in the YWA1-producing strain resulted in colonies that visually appeared more intensely orange after 3 days of cultivation as compared to the parent YWA1-producing *PKS12/npgA* strain (Figure 2). The yellow pigment observed in the plate medium surrounding *S. cerevisiae* colonies of the *PKS12/npgA* strain was no longer visible, suggesting conversion of YWA1 into another orange pigment, which did not readily escape the cells (Figure 2).

**Figure 4:**
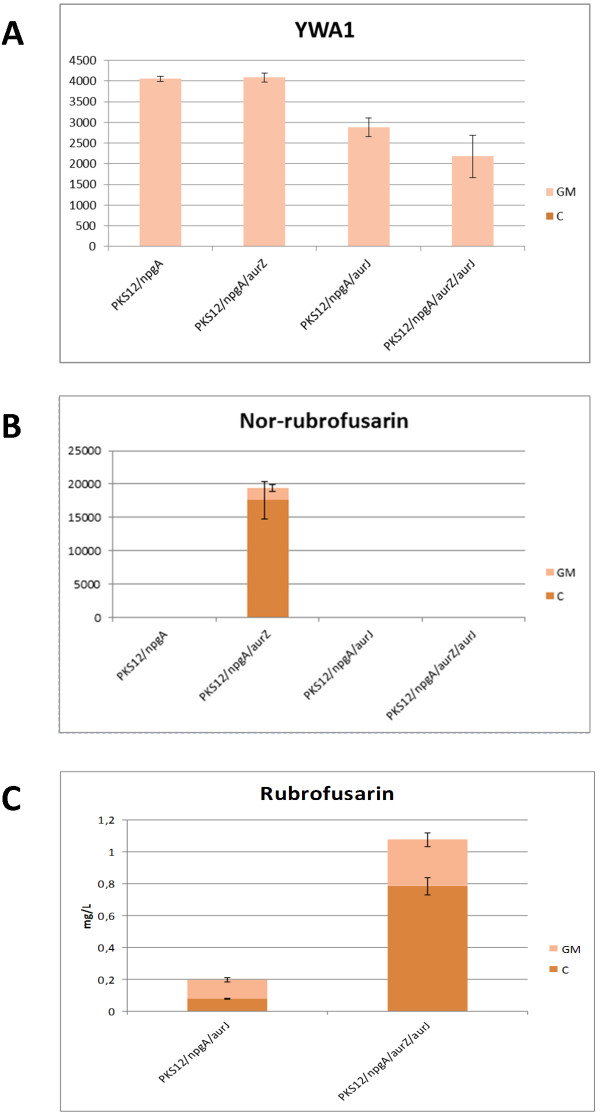
**Detection of intermediates inside and outside of the cells.** Detection of the rubrofusarin pathway intermediates in the constructed *S. cerevisiae* strains, shown by mean areas of the integrated peaks from extracted ion chromatograms for **A**) YWA1, **B**) Nor-rubrofusarin, and shown by mean titer (mg/L) calculated for **C**) Rubrofusarin. Measurements performed for the analyzed cell pellet (C) and growth medium (GM) of the constructed *S. cerevisiae* strains (see Table 1). Error bars indicate the std. deviation of each integrated peak (n = 3). Note that the mean areas are not comparable between different compounds.

Chemical analysis revealed a single new peak with a RT of 10.0 min and a [M + H]^+^ = 259.06 m/z which displayed a distinct UV absorption spectrum (see UV spectrum in Additional file 1). Based on a comparison to nor-rubrofusarin extracted from a nor-rubrofusarin-accumulating *F. graminearum ΔaurJ* reference strain, and by comparison to a previously published UV spectrum, the compound was identified as nor-rubrofusarin (Figure 3) [18]. Nor-rubrofusarin could be detected in both the cell pellet and growth medium, while YWA1 was still only detectable in the growth medium, further indicating that the observed change in colour is linked to the compounds of the identified peaks (Figure 4). A low level of nor-rubrofusarin was also detected in the cell pellet of the *PKS12/npgA* parent strain likely resulting from spontaneous conversion of YWA1. Spontaneous dehydration of YWA1 and YWA1-like compounds has previously been observed with purified compounds [18,26].

### Heterologous production of rubrofusarin

Introduction of *aurJ*, encoding an O-methyltransferase, into the *S. cerevisiae PKS12/npgA/aurZ* strain changed the visual phenotype to appear less orange during growth on plates (strain *PKS12/npgA/aurZ/aurJ*, Figure 2). Chemical analysis showed that nor-rubrofusarin was no longer accumulating. Instead, a new compound accumulated which had a RT of 13.3 min, [M + H]^+^ of 273.07 m/z and a unique UV absorption spectrum (see UV spectrum in Additional file 1). The compound was identified as rubrofusarin by comparison to rubrofusarin extracted from a rubrofusarin-accumulating *F. graminearum ΔaurF* reference strain (Figure 3) and by comparison to the previously published UV spectrum for rubrofusarin [18]. The rubrofusarin could be detected both in the extracted cell pellet and growth medium. YWA1 was still measurable in the growth medium, however at a reduced level (Figure 4A).

Surprisingly, introduction of *aurJ* in the *PKS12*/*npgA* strain resulted in colonies that were more orange than the *PKS12/npgA* strain which solely accumulates YWA1 (Figure 2). Chemical analysis showed the production of rubrofusarin, evidenced by the appearance of a peak at the same RT and [M + H]^+^ = 273.07 m/z as in the *PKS12/npgA/aurZ/aurJ* strain, but at an approx. six-fold lower level. These results are contrary to what has previously been observed in *F. graminearum ΔaurZ* mutants which only accumulate YWA1 even though they still express *aurJ*[18]. However, as we observed in the *PKS12/npgA* strain, low levels of YWA1 may undergo spontaneous conversion to nor-rubrofusarin, and subsequent methylation of this by AurJ may then explain the formation of rubrofusarin without the presence of AurZ. The difference in rubrofusarin concentration observed between the *PKS12/npgA/aurZ/aurJ* and *PKS12/npgA/aurJ* strains shows that AurZ is responsible for catalyzing the specific dehydration of YWA1 to yield nor-rubrofusarin, as previously suggested [18]. It is noteworthy that no methylated YWA1 was observed in the *PKS12/npgA/aurJ* strain at [M + H]^+^ = 291.08 m/z, suggesting that formation of rubrofusarin only proceeds through the nor-rubrofusarin intermediate, as previously proposed by Frandsen et al [12].

The presented heterologous production of rubrofusarin supports the biosynthetic pathway suggested by Frandsen *et al.* 2011 i.e. that PKS12, AurZ, and AurJ are stepwise responsible for formation of YWA1, nor-rubrofusarin, and rubrofusarin, respectively. In addition, it rules out the possibility that further, as yet unidentified enzymes are required for completion of the pathway.

The accumulated rubrofusarin from the strains *PKS12/npgA/aurZ/aurJ* and *PKS12/npgA/aurJ* was quantified by HPLC. Integration of rubrofusarin peaks from strain culture extracts showed a titer of 1.1 mg/L rubrofusarin produced by the *PKS12/npgA/aurZ/aurJ* strain, while 0.19 mg/L rubrofusarin was measured in the strain extract, when AurZ was excluded from the pathway.

In the present study no optimization strategies for production were employed. However, several simple optimization strategies would be applicable, such as increasing the gene copy number, tuning the individual expression level of pathway enzymes, and increasing the acetyl-CoA and malonyl-CoA precursor pools in the cell. In addition, optimizing the fermentation conditions is likely to substantially increase the titer. Production of cell pigmentation would provide a simple and convenient read-out for successful optimization.

The structurally simpler polyketide 6-MSA has previously been heterologously produced in *S. cerevisiae* reaching a titer of 1.7 g/L [6]. In another study, the 6-MSA titer was increased 60% by increasing the malonyl-CoA supply, via overexpression of the acetyl-CoA carboxylase ACC1, reaching a final 554 mg/L with their particular strain background [8]. These results suggest that the potential for rubrofusarin production in yeast may be significantly higher than what we detected in this pathway reconstruction study.

### *S. cerevisiae* as a heterologous host for fungal natural or synthetic polyketide pathways

Simple eukaryotic hosts, such as the unicellular yeast *S. cerevisiae,* hold great potential for expression and study of heterologous biosynthetic pathways, including those of entire gene clusters typical of secondary metabolism of filamentous fungi. In synthetic biology terms, *S. cerevisiae* represents a more “minimal host” than filamentous fungi, due to the absence of any significant endogenous secondary metabolites [27], which could otherwise result in pleiotropic effects, complicating product formation and the ensuing characterization of the heterologous pathways. Hence, a yeast-based system for heterologous expression of fungal polyketide biosynthetic pathways is very well-suited for efficient characterization of the continuously increasing pool of orphan fungal polyketide gene clusters being revealed by genome sequencing projects [28,29].

Reconstruction of the rubrofusarin pathway further opens the door for heterologous production of a diversity of related, but novel polyketides in *S. cerevisiae*, for example by co-expressing combinations of tailoring enzymes such as p450s, glycosyl-, acyl- and prenyl- transferases etc. selected from other secondary metabolite pathways and gene clusters. The genes of such enzymes could efficiently be laid “on top” of the rubrofusarin pathway for example by random assembly and expression on yeast artificial chromosomes [22]. The readily observable phenotype of the developed rubrofusarin-producing strain may also serve as a tool for optimizing yeast for general polyketide production, e.g. via enhanced precursor supply or screening for optimized enzymes such as PPTases.

## Conclusions

*S. cerevisiae* was engineered to produce the orange-brown polyketide pigment rubrofusarin, a common intermediate in a range of biosynthetic pathways found in different filamentous fungal species, by introduction of the three *F. graminearum* genes *PKS12*, *aurZ* and *aurJ* together with the PKS-activating PPTase gene *npgA.* A titer of 1.1 mg/L rubrofusarin was detected. The biosynthetic reconstruction further provides the evidence that PKS12 is responsible for formation of the pigment YWA1, whereas AurZ catalyzes the formation of the pigment nor-rubrofusarin from YWA1. The results confirm the previously proposed biosynthetic pathway for the formation of rubrofusarin using a bottom-up approach and show that no other enzymes are necessary. The study also demonstrates that it is possible to produce complex fungal polyketide products in *S. cerevisiae,* which opens up for the industrial production of a diversity of active and commercially interesting polyketides in the future.

## Materials and methods

### Strains

Molecular cloning was performed using *Escherichia coli* XL1 Blue, *E. coli* XL10 Gold (Stratagene) and *E. coli* DH5α (Invitrogen) competent cells. Final plasmids carried by strains in the chemical analysis are listed in Table 1. All *S. cerevisiae* strains constructed had the original genotype *mat α, trp1Δ, lys2Δ0, leu2Δ, his3Δ1, ura3Δ0, arg4::loxP* and were derived from *S. cerevisiae* BY4742 (Euroscarf). The constructed strains are listed in Table 1.

### Enzymes and oligonucleotides

All restriction enzymes were purchased from New England Biolabs. Oligonucleotides for subcloning and mutagenesis of *PKS12* were synthesized by Microsynth AG, while oligonucleotides for the USER friendly cloning were synthesized by Integrated DNA Technologies.

### Design and synthesis of genes

A wild type *F. graminearum* PH1 *PKS12* ORF was constructed using USER friendly cloning [30] by the assembly of the six exons predicted by MIPS [31] [FGDB: FGSG_02324] into the recipient vector BGHA-P1 [32] with *in vivo* ligation in *E. coli*. All exon fragments were PCR amplified from *F. graminearum* PH1 genomic DNA using primers designed with the PHUSER web tool (see oligonucleotides in Additional file 1) [33]. An internal HindIII restriction site in the ORF was silently mutated by mixing approx. 200 ng plasmid harboring the gene with 10 nmol of the primer Mut1Fwd and 10 nmol Mut1Rev in an otherwise standard 50 μL PCR mixture using iProof DNA polymerase and HF Buffer (BioRad) as the manufacturer specifies. The reaction was PCR thermocycled for 18 cycles followed by addition of 1 μL DpnI and 3 hours of incubation at 37°C to degrade the dam methylated original (non-mutated) plasmid strands. 2 μL reaction was transformed into 25 μL *E. coli* XL10 Gold competent cells and plasmids isolated with QIAprep Miniprep (Qiagen). A correctly mutated plasmid was subsequently validated by the absence of HindIII digestion.

The AurZ [FGDB: FGSG_02326] and AurJ [FGDB: FGSG_02325] protein sequences from *F. graminearum* PH1 were used for synthesizing the two gene ORFs with verification by sequencing and proprietary “GeneGPS” codon optimization for *S. cerevisiae* by DNA2.0. The *A. fumigatus npgA* gene [GenBank: XM_750100.1] was synthesized by DNA2.0 with codon-optimization for *S. cerevisiae* and verification by sequencing.

### Cloning of ORFs in pRS vectors

A set of pRS vectors [31] were used in which a unique AscI site had been inserted between the two PvuII sites, thus replacing the original multi-cloning site. All ORFs were subcloned with HindIII and SacII into vectors containing the respective, constitutive promoters and terminators (specified in Table 1) to form vectors with gene cassettes. The *PKS12* was provided with flanking HindIII and SacII restriction sites by PCR with iProof DNA polymerase using primers PKS12Fwd and PKS12Rev (see Additional file 1). All cassettes were subsequently cloned into the specified pRS plasmids (Table 1) with AscI. All ligations were performed using T4 DNA Ligase (New England Biolabs) and confirmed by sequencing (Microsynth). The pRS plasmids each harbour a unique auxotrophic marker gene (Table 1) and the ARS/CEN sequences for stable maintenance in *S. cerevisiae* at approx. one copy per cell, thus allowing strain-to-strain comparability. The *PKS12* ORF was controlled by the inducible *CUP1* promoter, which is induced from its low basal expression level by addition of Cu^2+^ to the medium [34].

### Transformation of *S. cerevisiae* strains

The *S. cerevisiae* strains (Table 1) were constructed by transformation with the specified plasmids (Table 1) according to the protocol described by Gietz & Schiestl, 2007 [35]. Transformants were plated on selective synthetic complete (SC) agar plates lacking the appropriate supplements uracil, histidine, tryptophan and leucine.

### Cultivation of the *S. cerevisiae* strains

The *S. cerevisiae* strains were grown for characterization on 2% agar plates with selective SC medium plus 500 μM CuSO_4_ for induction at 30°C. For chemical analysis, the strains were cultured by inoculating 5 mL selective SC medium from single colonies. These pre-cultures were incubated overnight at 30°C, with 160 rpm horizontal shaking. Main cultures of 10 mL selective SC medium, plus 300 μM CuSO_4_ for induction, were inoculated from the pre-cultures to an OD_600_ = 0.10 in three replicates of each pre-cultured strain. The main cultures were incubated for 96 hours at 30°C with 160 rpm horizontal shaking.

### Cultivation of reference *F. graminearum* strains

The reference *F. graminearum* strains *ΔaurZ*, *ΔaurJ* and *ΔaurF* were grown on Potato Dextrose Agar (Difco) plates at 20°C for 7 days.

### Chemical extraction and LC-MS analysis

Cells and growth medium of the main cultures were separated by centrifugation. The cells were resuspended in 1 mL dH_2_O and extracted by vigorous shaking with 5 mL extraction solvent (methanol:dichloromethane:ethyl acetate (1:2:3) + 1% formic acid). The mixtures were sonicated for 1 hour in an ultrasonic bath, centrifuged, filtered through filter paper and dried in a SpeedVac. Eight mL of the growth medium were extracted by vigorous shaking with 10 mL extraction solvent after which the organic phases were dried in a SpeedVac. Three 10 mm plugs were cut out of the mycelium of each reference *F. graminearum* strain. The plugs of each replicate were mashed in 10 mL ethyl acetate and shaken vigorously to facilitate extraction. The organic phase was recovered following centrifugation and filtering, at which point it was dried in a SpeedVac. The extractions from the *F. graminearum* reference strains were performed in triplicates.

All samples were reconstituted in 150 μL dimethyl sulfoxide. Five μL was injected and analyzed by LC-UV/ELSD on a Waters Acquity UPLC (Waters Corporation, Milfords, MA, USA), running a gradient of mobile phases A (water + 0.1% formic acid) and B (acetonitrile +0.1% formic acid), going from 1 to 100% B in 15 min, flow rate of 0.4 mL/min. The column was a Waters Acquity BEH C18 (2.1 mm × 100 mm × 1.7 μm (Waters Corporation, Milfords, MA, USA) temperature-controlled at 35°C. Liquid chromatography was coupled to a Bruker Daltronics microQTOF II (Bruker Daltonics, Bremen, Germany) using electrospray ionization source in positive mode: End plate Offset: -500 V, Capillary voltage: 4500 V, Nebulizer: 1.6 Bar, Dry Gas: 8.0 L/min, Dry temperature: 180°C. Extracted ion chromatograms for YWA1 (m/z 277.0707 ± 0.01), nor-rubrofusarin (m/z 259.0601 ± 0.01) and rubrofusarin (m/z 273.0753 ± 0.01) were created using the Bruker DataAnalysis 4.0 software. The integration of the peaks was performed with the DataAnalysis Integrate functions (with default settings).

### Quantification of rubrofusarin production

Cells and growth medium from 8 mL main culture were separated by centrifugation. The cell pellet was resuspended in 1 mL dH_2_O and 5 mL ethyl acetate. The cell pellet solution was sonicated for 30 min. in an ultrasonic bath followed by vigorous shaking. The growth medium supernatant was shaken vigorously with 8 mL ethyl acetate. The ethyl acetate phases of growth medium and cell pellet were collected individually and allowed to evaporate in a fume hood. The extracted compounds of growth medium and cell pellet were reconstituted in 1.5 mL and 1.0 mL methanol, respectively. A rubrofusarin standard, purified from *F. graminearum,* was purchased from BioViotica Naturstoffe GmbH (Liestal, Switzerland) and reconstituted to 1 mg/mL in dimethyl sulfoxide. A standard curve was prepared by dissolving the rubrofusarin standard in methanol in a range from 4 μg/mL to 10 μg/mL. Twenty μL of each standard concentration and the extracted samples were analyzed by HPLC using an Ascentis C18 column (particle size: 5 μm, length × I.D.: 15 cm × 4.6 mm) (Sigma-Aldrich) running a gradient of mobile phases C (10 mM ammonium formate pH 3.0) and D (acetonitrile) at a flow rate of 1 mL/min, starting at 25% D (0-3 min), going to 80% D (3-15 min), remaining at 80% D (15-16 min), going to 25% D (16-18 min) and remaining at 25% D (18-20 min).

## Competing interests

PR, UM and RF declare no competing interests. MN is employed by Evolva SA.

## Authors’ contributions

PR and MN planned the experimental procedures of the study. PR carried out the experiments, except for cloning the exons of *PKS12*, which RF did. PR analyzed and interpreted the data and wrote the manuscript together with RF, MN and UM. All authors read and approved the final manuscript.

## Supplementary Material

Additional file 1 Oligonucleotides, UV spectra and HPLC chromatograms. Click here for file
