# Supplementary material for: Reconstruction of the biosynthetic pathway for the core fungal polyketide scaffold rubrofusarin in Saccharomyces cerevisiae
Source: Microb Cell Fact. 2013 Apr 4;12:31. doi: 10.1186/1475-2859-12-31 (PMC3654996; doi:10.1186/1475-2859-12-31)

Supplemental data:
Oligonucleotides used in the study

Name	Sequence (5' to 3')	Purpose
**Mut1Fwd**	TTCAACAGAAGCTATTTCCAAA	HindIII removal
**Mut1Rev**	TTTGGAAATAGCTTCTGTTGAA	HindIII removal
**PKS12Fwd**	TGTAAGCTTATGGAGGTATTCGTTTTTGG	HindIII site add
**PKS12Rev**	TATCCGCGGCTAATTGTCCAAGGCCCG	SacII site add
**PKS12-Exon1-F**	GGGTTTAAUATGGAGGTATTCGTTTTT	USER Fusion
**PKS12-Exon1-R**	AAAGTGAAUGAAACTGCAAAGATGGTA	USER Fusion
**PKS12-Exon2-F**	ATTCACTTUTACGATGGACAAGGTCGT	USER Fusion
**PKS12-Exon2-R**	AGTCCCATUTCAATGATGAATGAGTCAAGTCG	USER Fusion
**PKS12-Exon3-F**	AATGGGACUTGCTCGATCGTCAGTTCC	USER Fusion
**PKS12-Exon3-R**	ACCAGGAAUAAAATAAGTGTCGACATTCTGC	USER Fusion
**PKS12-Exon4-F**	ATTCCTGGUGGCAACAGAGCGTTCACT	USER Fusion
**PKS12-Exon4-R**	AGTTGCCGGUTCTTGATAGGAAGTGGCCTCG	USER Fusion
**PKS12-Exon5-F**	ACCGGCAACUGCAACACCTTTGACGATG	USER Fusion
**PKS12-Exon5-R**	AATGTTAUGTCTCCATAAACACCAACC	USER Fusion
**PKS12-Exon6-F**	ATAACATUCCAAGGTTTGCCGCGACG	USER Fusion
**PKS12-Exon6-R**	GGTCTTAAUCTAATTGTCCAAGGCCCGAGC	USER Fusion

UV spectra

UV spectrum for rubrofusarin obtained from growth medium of *S. cerevisiae* *PKS12/npgA/aurZ/aurJ*. RT = 13.33 min

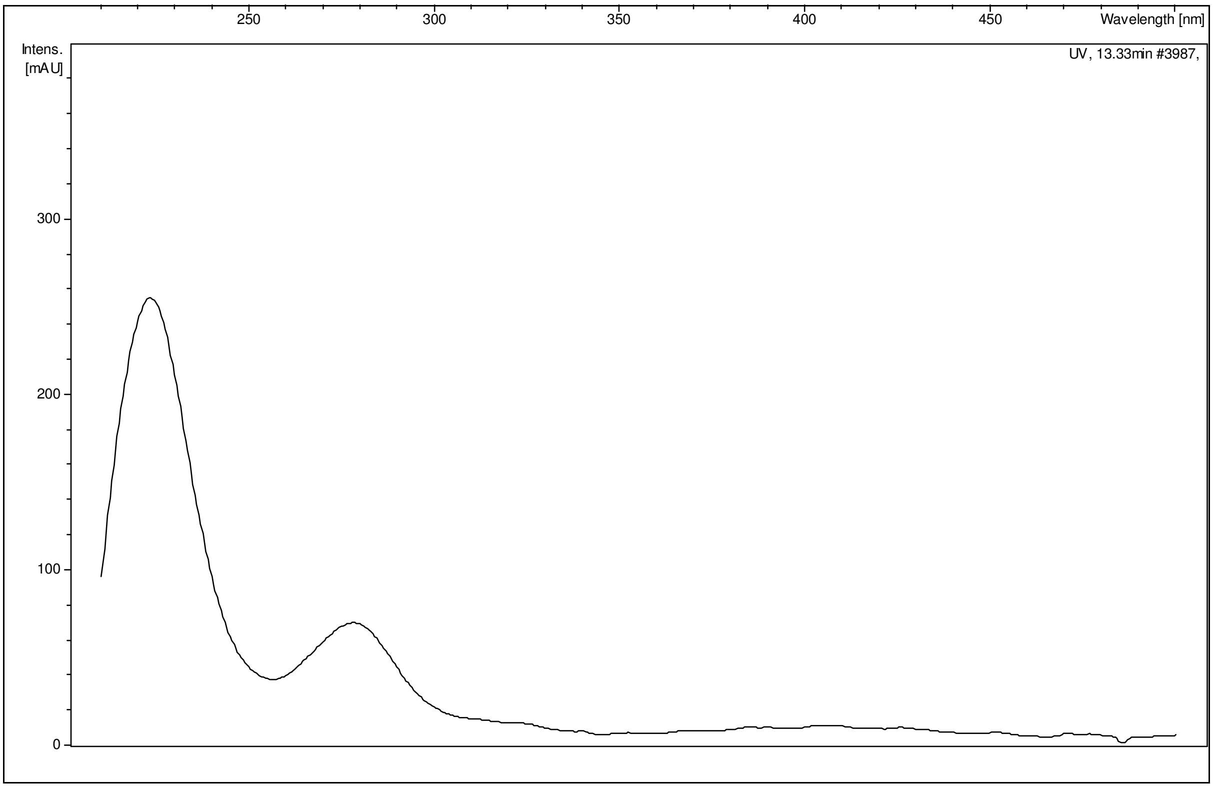

UV spectrum obtained from growth medium of the control *S. cerevisiae.* RT = 13.33 min.

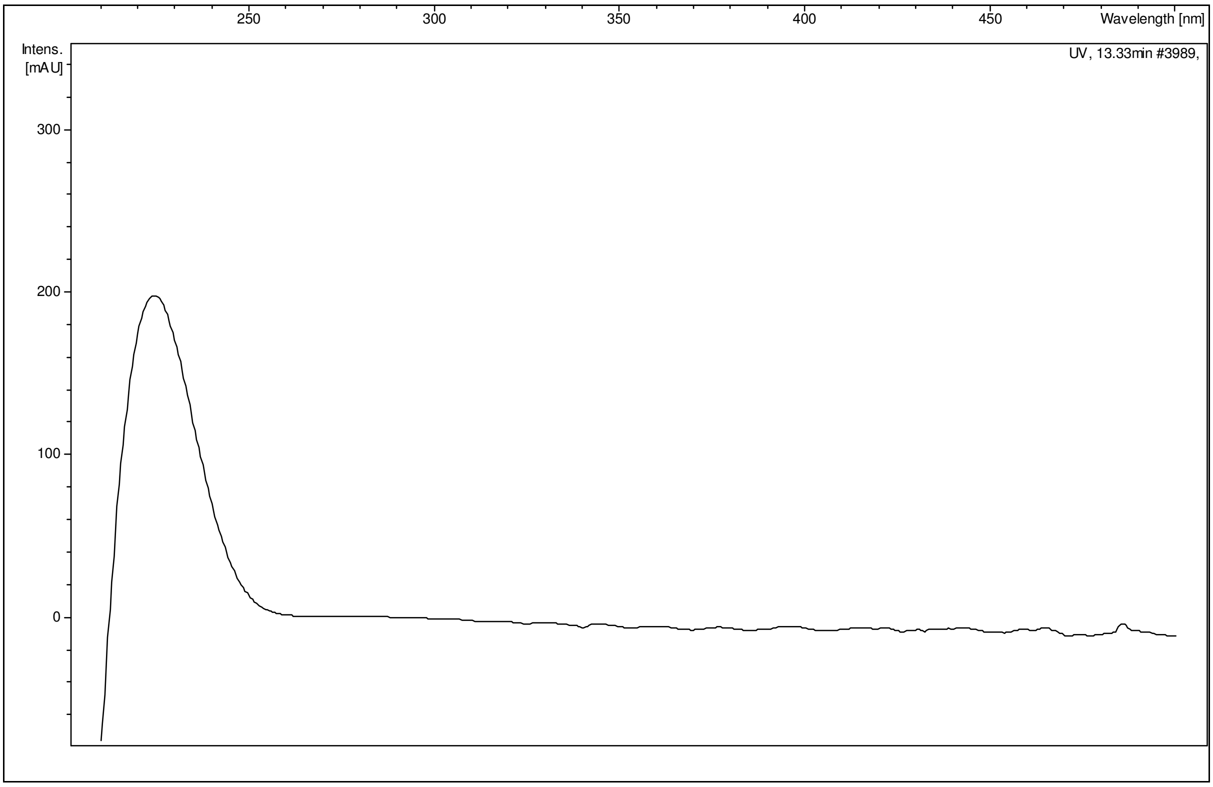

UV spectrum for nor-rubrofusarin obtained from cell pellet of *S. cerevisiae*

PKS12/npgA/aurZ. RT = 10.00 min

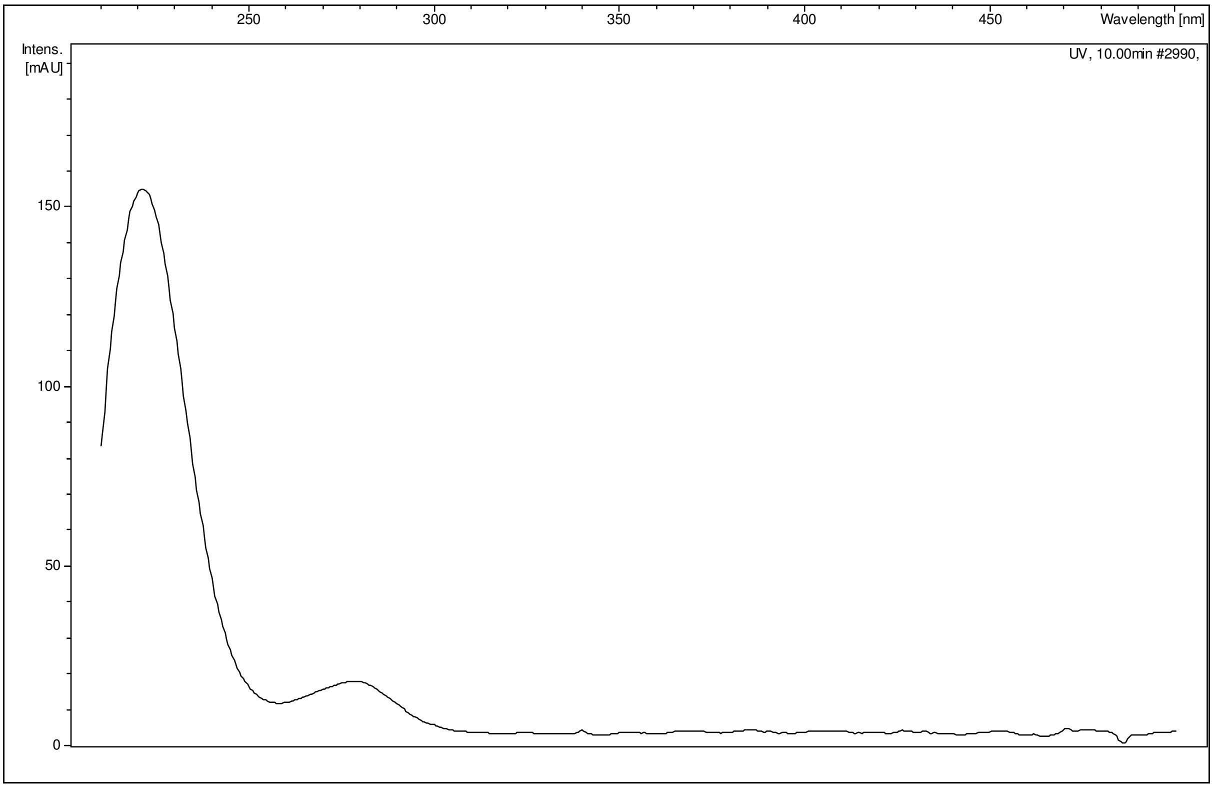

UV spectrum obtained from cell pellet of the control *S. cerevisiae*. RT = 10.00 min.

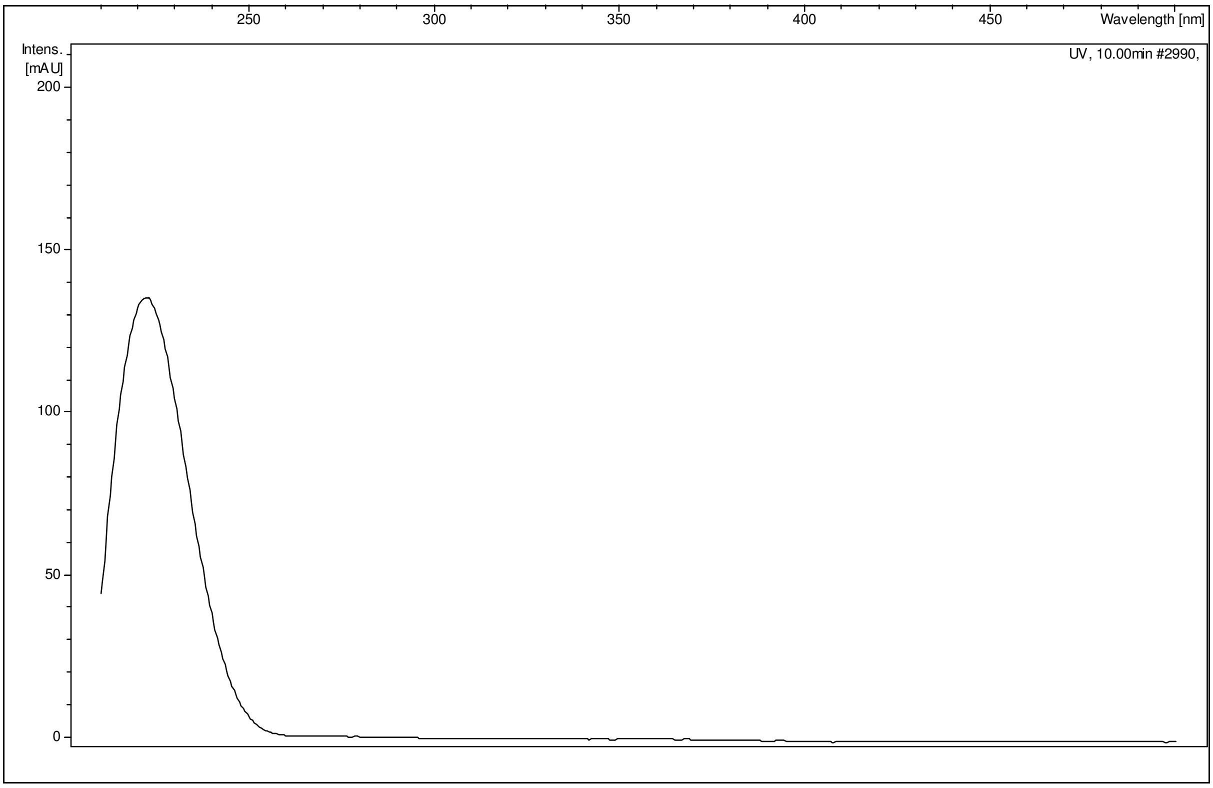

HPLC chromatograms

Chromatogram at 279 nm, obtained for a rubrofusarin standard and the *S. cerevisiae* strain *PKS12/npgA/aurZ/aurJ* extracted from growth medium and cell pellet, respectively (from top down). Rubrofusarin was eluted at RT = 15.3 min.

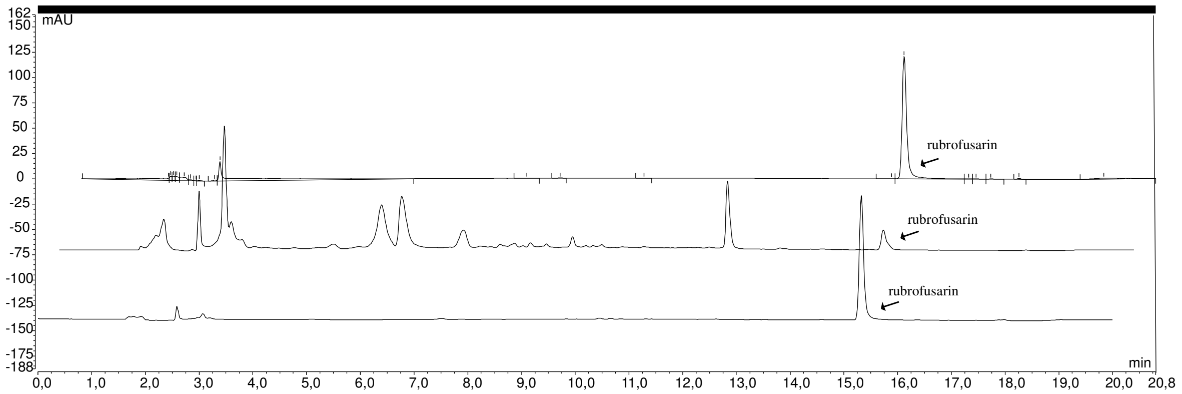

Chromatogram at 279 nm, obtained for a rubrofusarin standard, and the *S. cerevisiae* strain *PKS12/npgA/aurJ* extracted from growth medium and cell pellet, respectively (from top down). Rubrofusarin was eluted at RT = 15.3 min.

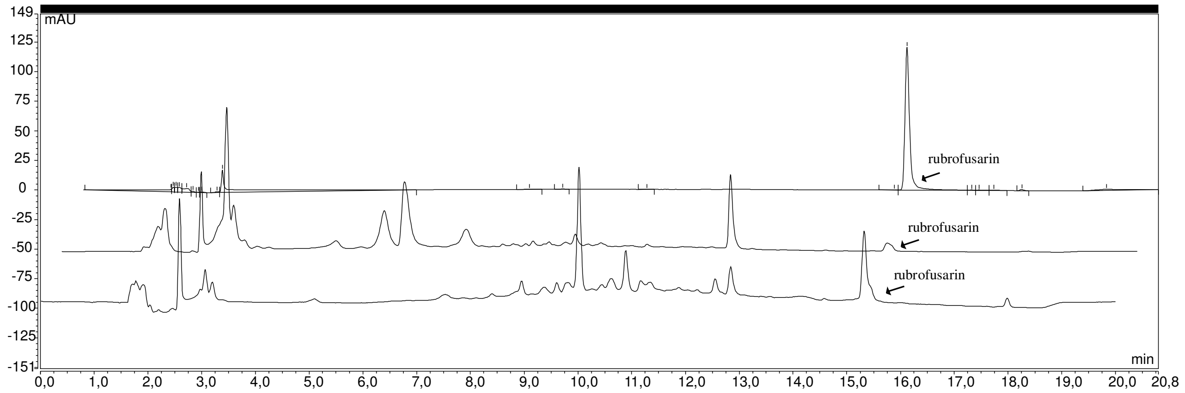

Standard curve with linear regression calculated for the relation between rubrofusarin concentration (mg/L) and the rubrofusarin peak area

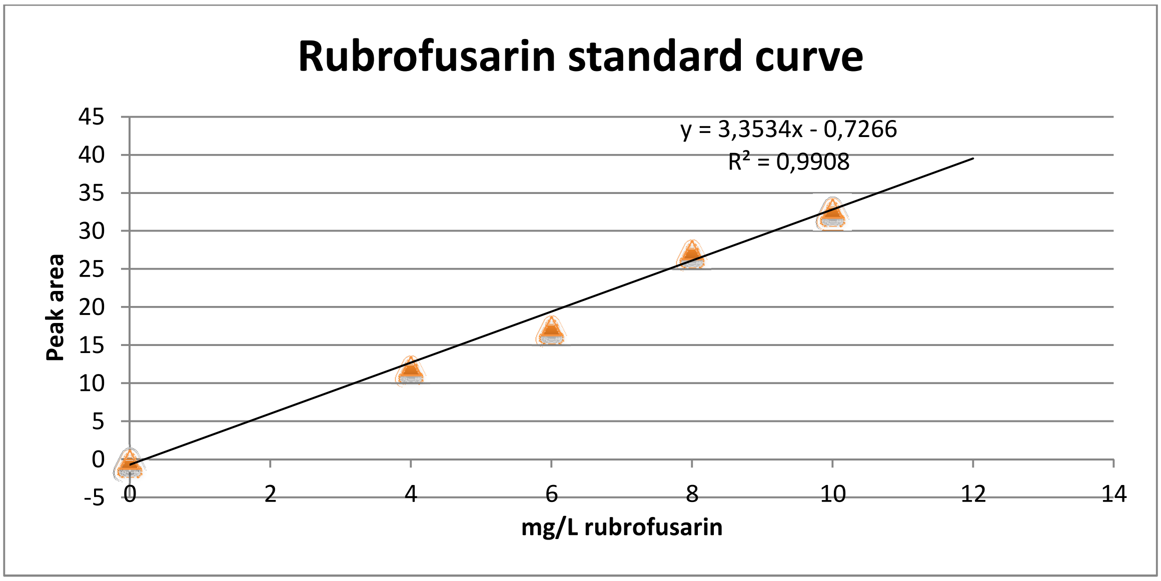

Supplement: Additional file 1 — Oligonucleotides, UV spectra and HPLC chromatograms. [file 1475-2859-12-31-S1.pdf]
